# Evaluation of a school-based multicomponent behavioural intervention in deprived urban areas for children classified as overweight, obese or at risk for overweight

**DOI:** 10.1186/s13104-021-05513-y

**Published:** 2021-03-17

**Authors:** Gitte C. Kloek, Martien W. A. Jongert, Sanne I. de Vries

**Affiliations:** 1 Research Group Healthy Lifestyle in a Supporting Environment, Faculty of Health, Nutrition & Sport, The Hague University of Applied Sciences, PO Box 13336, 2501 EH The Hague, The Netherlands; 2 Dutch Institute of Allied Health Care, PO Box 1161, 3800 BD Amersfoort, The Netherlands

**Keywords:** Overweight, Obesity, Children, Body mass index, Prevention, Multicomponent behavioural intervention

## Abstract

**Objective:**

This study evaluated the effect of an after-school group-based medium-intensity multicomponent behavioural intervention programme for children aged 8–12 years classified as overweight, obese or at risk for overweight on body mass index standard deviation score (BMI SDS). In accordance with standardized protocols body weight and height were measured in 195 participants (88 boys, 107 girls) at baseline and at the end of the programme. A total of 166 children derived from a school-based monitoring system served as control group. Multivariate regression analyses examined the effect of the intervention and the independent factors associated with better outcomes in the intervention group.

**Results:**

Analysis of covariance showed a significant intervention effect on BMI SDS in favour of the intervention group (b-coefficient − 0.13 ± 0.03; p < 0.01) compared with the control group. Change in BMI SDS between baseline and follow-up in the intervention group was associated with baseline age (b-coefficient 0.03 ± 0.02; p = 0.04) but was independent from gender, ethnicity, baseline BMI SDS, time between baseline and follow-up, school year and attendance rate.

## Introduction

Worldwide the growing incidence and prevalence of childhood obesity and overweight is a reason for concern [1]. Since 1980, the prevalence rates of childhood overweight have increased two to three fold in The Netherlands [2]. In 2009, 13–15% of the Dutch children were classified as overweight and 2% were obese [2], according to the International Obesity Task Force (IOTF) cut off values [3]. Although the prevalence of childhood overweight appears to be stabilizing at different levels in different countries it is still a public health issue, especially when ethnicity is taken into account [4]. In the Netherlands, children with backgrounds originating from Turkey and Morocco are at the highest risk: between 27 and 30% are overweight and between 6.5% and 8.4% are obese [5]. In The Hague, the third largest city in The Netherlands, an integrated approach for the prevention of childhood obesity was set up in 2006. The approach integrates a system of early identification and referral of children with overweight or obesity to appropriate care and support within multiple settings such as the community, schools, or paediatric primary care [6]. Behavioural interventions for treating overweight in children that combine changes in diet, physical activity and behavioural management strategies are promising means to lower body mass index standard deviation scores (BMI SDS) in participants [7–11]. According to Whitlock et al. [9] medium- (26 to 75 h) to high- (more than 75 h) intensity behavioural interventions have a significantly larger effect on weight outcomes than very low-intensity (under 10 h) interventions. The aim of the present study was to evaluate whether the WOWIJS programme, an after-school group-based medium-intensity multicomponent behavioural intervention in deprived urban areas for children classified as overweight, obese or at risk for overweight, would be effective in preventing further increases in BMI SDS.

## Main text

### Methods

#### Intervention programme

Since 2010, the WOWIJS programme, Dutch acronym for ‘**W**ork**O**ut** W**at** I**s** J**ouw** S**tijl’ [Work Out What Is Your Style] is an ongoing after-school group-based multicomponent behavioural intervention for children aged 8–12 years with overweight, obesity, or at risk for overweight in The Hague, The Netherlands [12]. The intervention serves primary schools in more deprived areas of the city. Relatively many families in these deprived areas have low incomes and originate from four of the major ethnic groups in the Netherlands [13]. The children participate in weekly 90-min group sessions that include exercise training, nutrition education and lifestyle counselling during the school year. WOWIJS is located in the child’s immediate environment, either at their own school or in a sports hall near their school. The group sessions are carried out by a team of professionals: a dietician, a physical education (PE) teacher, and a child psychologist.

#### Design and study population

The WOWIJS intervention programme is practice-based and was not designed as a controlled trial. In 2011 and 2012, the identification of children who were likely to benefit from the WOWIJS intervention programme was done by the school’s PE teacher. Children with overweight, obesity without co-morbidities or children with a high risk of overweight in the near future were eligible for WOWIJS. Children were classified as ‘high risk for overweight’ if there was a known family history of overweight or obesity in older siblings. In 2011, 152 children from five primary schools and in 2012, 126 children from six primary schools started in the WOWIJS programme. Depending on the start date, the children took part in 30 to 50 group sessions during the school year. Baseline and follow-up measures were available from in total 195 (66%) out of 296 children. A logistic regression analysis showed that lost to follow-up was associated with a lower attendance rate of the program (p < 0.01). To evaluate the effect of the intervention, this study compared children in the intervention programme with overweight or obese children who were selected from a school-based monitoring system to follow developments in (over)weight in school children in The Hague. For the monitoring system, the PE teacher collected annual data on children’s weight and height according to a standardized protocol. This study used data that were collected in 2011 en 2012 among 166 children who attended a primary school not participating in the WOWIJS programme but that was situated in an area with a comparable deprivation score, who were either overweight or obese and aged between 8 and 12 years in 2011 and who had participated in a follow-up measurement in 2012.

#### Measures

Demographic data were collected at baseline and attendance data for the group sessions were collected throughout the programme. Country of birth of the participants’ parents determined the categorization by ethnic group. An attendance list was filled out for every group session to determine the overall attendance rate at the end of the programme. Anthropometric data were collected at the beginning and at the end of WOWIJS. Body weight (to the nearest 0.1 kg) and height (to the nearest 0.1 cm) were measured by a trained WOWIJS dietician using the same standardized protocols as in the monitoring system. Body weight was measured without heavy clothing and shoes. Body mass index (BMI, kg/m^2^) was calculated as weight/height^2^. Overweight and obesity prevalence rates were defined according to the extended International Obesity Task Force (IOTF) cut-off points and gender- and age-specific BMI SDS was calculated using the least square means coefficients corresponding to these IOTF cut-off points as a reference [14]. Change in BMI SDS at the end of the programme was calculated as BMI SDS at follow-up minus baseline BMI SDS. Participants were divided in four subgroups on the basis of their change in BMI SDS: BMI SDS increased, BMI SDS decreased by > 0 to < 0.25, BMI SDS decreased by ≥ 0.25 to < 0.5 and BMI SDS decreased by ≥ 0.5. These groups were chosen on the basis of previous studies where a minimum reduction in BMI SDS of at least 0.25 was required to improve body composition and cardio metabolic health [15, 16].

#### Statistical analyses

Baseline characteristics and outcomes at follow-up of the children in the intervention group were compared with the control group. Chi-squared tests were used for categorical variables and t-tests were used for continuous variables. To estimate the effectiveness of WOWIJS on change in BMI SDS, a mixed model analysis of covariance with baseline value of BMI SDS, gender, age and time between baseline and follow-up measurement as covariates, was applied. The independent factors associated with better outcomes in WOWIJS participants were assessed using a two-level multilevel linear model to account for data clustering at the school level. All P values reported are two-sided with the significance level set to 0.05. Statistical analyses were performed using SAS 9.4 (SAS Institute, Cary, NC, USA).

### Results

Table 1 shows the characteristics of the study population. Age, BMI, weight status and evaluation period were significantly different between the intervention and control group. WOWIJS participants followed on average 27 (± 9) group sessions, with a minimum of 5 and a maximum of 49 lessons. The average attendance rate of the WOWIJS group sessions was 84% (± 13%).

**Table 1:** Characteristics of the intervention group and school-based control group at baseline

Characteristic	Intervention group (N = 195)	School-based control group (N = 166)	p-value
Gender (girls)	107 (55)	91 (55)	0.99
Age (years)	9.7 (1.2)	9.4 (1.1)	0.01
Ethnicity^a^			
Dutch	3 (2)	NA	
Surinamese	64 (35)	NA	
Moroccan	49 (27)	NA	
Turkish	40 (22)	NA	
Other	25 (14)	NA	
BMI (kg/m^2^)	23.0 (3.2)	22.4 (2.9)	0.03
BMI SDS	2.05 (0.58)	1.99 (0.50)	0.32
Weight status			< 0.01
Normal weight^b^	21 (11)	0	
Overweight	98 (50)	116 (70)	
Obese	37 (39)	50 (30)	
Evaluation period (days^c^)	196 (54)	306 (77)	< 0.01

Data as mean (SD) or N (%)

NA not available for the school-based control group

^a^Missing data n = 14 participants

^b^Children at high risk for overweight in the near future

^c^Number of days between baseline and follow-up

Comparing anthropometric changes at follow-up demonstrated a significantly smaller BMI increase and a greater BMI SDS reduction in the intervention group compared with the control group (Table 2). Analysis of covariance showed a significant intervention effect on BMI SDS in favour of the intervention group (b-coefficient − 0.13 ± 0.03; p < 0.01).

**Table 2:** Anthropometric changes from baseline to follow-up in the intervention group and school-based control group

	Intervention group (N = 195)	School-based control group (N = 166)	p-value^a^
	Mean (SD) difference or N (%)	Mean (SD) difference or N (%)	
Change in BMI (kg/m^2^)	0.10 (0.88)	0.69 (1.40)	< 0.01
Change in BMI SDS	− 0.13 (0.22)	− 0.06 (0.26)	0.02
BMI SDS reduction			0.08
None Δ BMI-SDS > 0	60 (31)	72 (43)	
Minimal Δ BMI-SDS ≤ 0 ≤ − 0.25	84 (43)	63 (38)	
Successful Δ BMI-SDS ≤ − 0.25 ≤ − 0.5	38 (19)	22 (13)	
Highly successful Δ BMI-SDS ≤ − 0.50	13 (7)	9 (5)	

Follow-up measurement intervention group at the end of the program; follow-up measurement school-based control group in the next school year

^a^Unpaired t-test or chi-squared test

The multilevel model showed that the change in BMI SDS between baseline and follow-up in the intervention group was only associated with baseline age (b-coefficient 0.03 ± 0.02; p = 0.04) and was independent from gender, ethnicity, baseline BMI SDS, duration of follow-up, school year and WOWIJS attendance rate (Table 3). The variance attributable to school location was 5.3%.

**Table 3:** Independent factors and change in BMI SDS in the intervention group: multilevel regression analysis (N = 181)

Independent factors	Beta	95% CI	p-value
Gender			
Girls vs boys	− 0.01	− 0.08 to 0.07	0.86
Age (years)	0.03	0.001 to 0.06	0.04
Ethnicity			
Surinamese vs Moroccan	− 0.01	− 0.15 to 0.13	0.86
Surinamese vs Turkish	− 0.01	− 0.15 to 0.13	0.88
Surinamese vs Other	0.02	− 0.10 to 0.14	0.76
BMI SDS baseline	0.02	− 0.04 to 0.08	0.49
Evaluation period (days^a^)	− 0.0004	− 0.0001 to 0.0001	0.38
School year			
2012 vs 2011	0.03	− 0.05 to 0.10	0.47
Attendance rate (%)	− 0.11	− 0.40 to 0.18	0.45
Intraclass Correlation Coefficient school level: 0.053			

Mutually adjusted associations with change in BMI SDS in the intervention group, missing data n = 14 participants

^a^Number of days between baseline and follow-up

### Discussion

This study showed that the WOWIJS intervention improved BMI SDS in participants. WOWIJS participants had a more favourable BMI SDS reduction distribution compared with children in the school-based control group. Although the decrease in BMI SDS in the WOWIJS intervention group was modest, it is in accordance with findings from a recent meta-analysis that showed that behavioural interventions for the treatment of overweight or obese children from the age of 6 to 11 years demonstrated a decrease in BMI SDS of 0.02 to 0.10 compared with a no treatment group [17]. Furthermore younger children had better outcomes on change in BMI SDS than older children. Reinehr et al. also reported better reduction of BMI SDS in younger children after participation in a 1-year lifestyle intervention [18]. An explanation may be the social context that contributes to developing healthy lifestyles over time. Older children may experience more peer pressure for unhealthy behaviours than younger children [19] and therefore achieve less BMI SDS reduction.

Our study possesses several strengths, including a multicomponent behavioural intervention designed according to evidence-based recommendations. These kind of interventions are efficient when it comes to paediatric obesity [9]. Overall group session attendance was high, therefore the received intervention dose of most participants meets the dose of a medium intensity behavioural intervention. Easy access to the intervention in the child’s own school environment after school hours may have contributed to these high attendance rates. Furthermore the lost-to-follow up rate of 34% was lower than the up to 43% reported in a meta-analyses for randomized controlled trials that report dropout rates in paediatric obesity interventions [7].

In conclusion, BMI SDS in an ethnic diverse group of children classified as overweight, obese or at risk for overweight can be improved by participating in a school-based medium-intensity multicomponent behavioural intervention. Without the programme participants BMI SDS may continue to increase over time. The WOWIJS programme is a secondary obesity prevention programme and therefore fills a gap between primary prevention and tertiary preventive care treatment programs for obese children. The programme represents a realistic approach for the prevention of childhood obesity in a hard to reach populations in deprived urban areas. The ongoing WOWIJS programme now includes a smartphone application to stimulate physical activity and healthy food choices at home. This application may be attractive to the older participants in the programme and therefore it may increase their change on better outcomes. A new study should investigate the effectiveness of the extended programme on BMI SDS but also the long-term effects to determine the sustainability of the outcomes.

## Limitations

- Long-term outcomes on change in BMI SDS after the end of the intervention were not assessed.
- The school-based control group was not an exact match for the intervention group. Children were somewhat younger which may also explain the lower BMI at baseline.
- Several children with a normal weight status were included in the intervention group because they were at high risk of overweight in the near future. They could not be matched with normal weight children in the monitoring system.
- It is unknown whether the overweight children in the school-based control group participated in other behavioural interventions.
- The follow-up period was shorter in the intervention group compared with the control group. However the length of the evaluation period was not associated with changes in BMI SDS.

## Data Availability

The data that support the findings of this study are not publicly available due to the original consent and ethics approval not containing approval from the participants in the WOWIJS intervention programme for data sharing. Data are however available from the authors upon reasonable request and with permission of the SHJG Foundation (non-profit foundation for youth health promotion in the Hague, The Netherlands).

## Abbreviations

BMI: Body mass index
BMI SDS: Body mass index standard deviation score
IOTF: International obesity task force
WOWIJS: Dutch acronym for '**W**ork**O**ut** W**at** I**s** J**ouw** S**tijl' [Work Out What Is Your Style]
